# Editorial: Dysfunction and Repair of Neural Circuits for Motor Control

**DOI:** 10.3389/fnmol.2021.669824

**Published:** 2021-03-22

**Authors:** Andrew Paul Tosolini, George Z. Mentis, John H. Martin

**Affiliations:** ^1^Department of Neuromuscular Diseases, UCL Queen Square Institute of Neurology, University College London, London, United Kingdom; ^2^Department of Pathology & Cell Biology and Neurology, Center for Motor Neuron Biology and Disease, Columbia University, New York, NY, United States; ^3^Department of Molecular, Cellular and Biomedical Sciences, Center for Discovery and Innovation, City University of New York School of Medicine, New York, NY, United States

**Keywords:** motor neurons, sensory neurons, regeneration, spinal cord injury, ALS, SMA, inflammation, glia

The dissolution of normal behavior in pathological conditions, after trauma, or in neurodegenerative diseases is often attributed to the selective dysfunction and degeneration of particular classes of vulnerable neurons. However, vulnerable neurons are embedded in neuronal circuits that often play major roles in the progression of the pathophysiological state, by cell-autonomous and non-cell autonomous mechanisms. For example, voluntary motor behavioral impairments occur after brain or spinal cord injury because descending projection neurons are vulnerable to axonal damage and are unable to regenerate anew. This limitation reflects both a loss of intrinsic axon regenerative capacity in mature neurons and extrinsic regulation by non-neuronal cells. Similarly, motor neurons deprived of supraspinal inputs or in neurodegeneration (e.g., ALS, SMA) undergo an array of well-described molecular events (Schwab and Bartholdi, 1996; Brown and Al-Chalabi, 2017; Groen et al., 2018) that also include morphological remodeling (Bose et al., 2005; Dukkipati et al., 2018). If therefore, repair or restoration of normal function is sought after, a clear understanding is required for the molecular, cellular, and neuronal circuit mechanisms involved. Over the last decade, several significant advances in our understanding of the development and operational principles of neural circuits demonstrate the complexity of the central nervous system under normal conditions and the diverse mechanisms that lead to dysfunction.

Everyday actions in essential complex behaviors such as walking, feeding, and breathing, require the specific integration of neural circuits that flawlessly operate with precision, co-ordination, and synchrony (Arber, 2012). As summarized in Figure 1, for voluntary movement to occur, the motor cortical areas must initiate communication with the spinal cord circuitry, which in turn convey these commands *via* spinal motor neurons to the skeletal muscles. Equally important, sensory information from the periphery is essential for the proper activation and function of neural circuits involved in motor control. Intrinsic to these processes are the influences of spinal excitatory and inhibitory interneurons.

**Figure 1:**
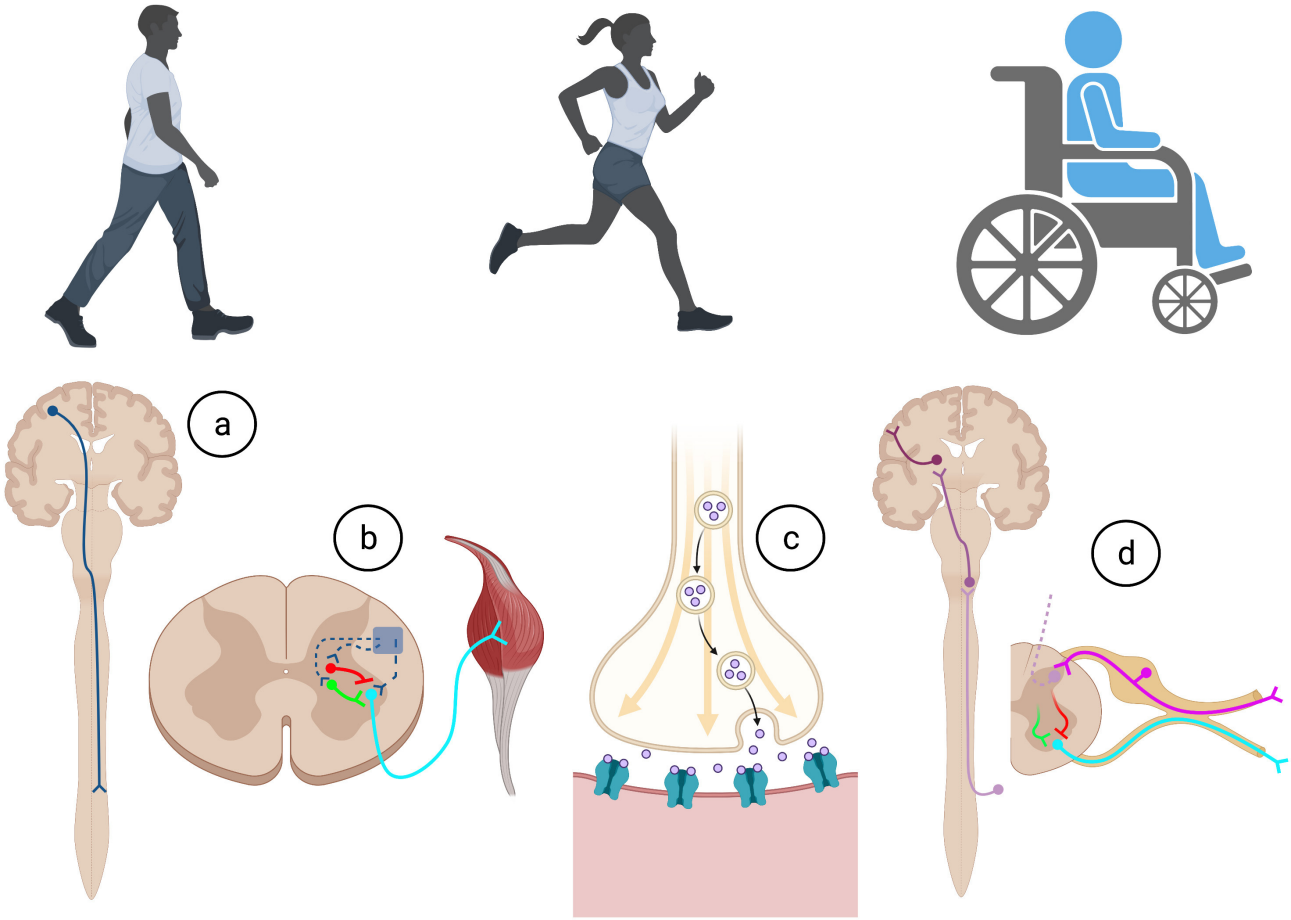
Execution of all movements in health and after trauma require a complex series of co-ordination of cellular and molecular actions involved within the **(A)** descending motor system, **(B)** intrinsic spinal cord circuitry, **(C)** neuromuscular junction, and **(D)** afferent feedback through the ascending sensory system. Created with BioRender.com.

However, after injury or in disease states, these neural circuits may be affected in subtle ways but over time may result in a multitude of effects causing dysfunction; not only in vulnerable neurons but also by disrupting the signaling and connectivity of integrated neural circuits. As neural circuits are disrupted through trauma [spinal cord injury (SCI), peripheral nerve injury, stroke] or in neurodegenerative diseases [amyotrophic lateral sclerosis (ALS), spinal muscular atrophy (SMA)], determining the timing and primacy of the earliest pathological events are critical questions to be addressed if normal function is to be restored. Uncovering therefore the initiating pathogenic events are critical steps in proposing potential therapeutic avenues.

However, a paramount challenge for the field is to harmonize and integrate effectively the increasing number of studies that separately focus on molecular events in single cells (e.g., transcriptomics, proteomics) or on cellular phenotypes in neurons and glia (e.g., neuronal dysfunction, neurodegeneration, synaptic plasticity, astrocytic, or microglia proliferation) (Stuart and Satija, 2019). To this end, an exciting challenge is to decipher adaptive from maladaptive molecular and cellular changes within individual neurons and their supporting cells and the influence they exert on motor circuits after neural trauma (Ilieva et al., 2009; Eroglu and Barres, 2010; Huntley, 2012). We launched this Research Topic to provide a platform to encourage discussion on recent advances in knowledge and/or therapeutic tools to investigate motor circuitry following certain pathological conditions.

The topic on *Dysfunction and Repair of Neural Circuits for Motor Control* is devoted to neuronal circuits involving cortical, spinal, and peripheral neurons in healthy and pathological conditions. The articles in this collection—comprising five original studies, three extensive-reviews and two mini-reviews—explore how certain circuits regulate and integrate their actions, how they are altered in disease or after trauma, and efforts to repair circuits to restore normal function. Discussions span human and animal models of impaired motor circuits and a focus that includes much of the neuraxis: the motor cortex, hindbrain, spinal cord, somatic sensory neurons, and peripheral nerves.

The five original studies traverse the neuroanatomical spectrum, spanning hindbrain neurons, upper motor neurons, and sensory neurons to glial-peripheral nerve interactions, with particular focus on regeneration, inflammation, and excitability in ALS animal models (Table 1). Huang et al. study the modulation of miR-133b to promote axon regeneration of Mauthner-cells in zebrafish hindbrain. Ballou et al. characterize the inflammatory and glial response to cortical transplants after injury. Chen et al. reveal the involvement of Schwann cells in peripheral nerve regeneration. Zeng et al. describe inflammation cascades in DRG sensory neurons. Jara et al. describe circuitry changes in the motor cortex of pre-symptomatic ALS mice.

**Table 1:** Highlights from the original research published within this Research Topic.

**Paper**	**Purpose**	**Highlights**	**Model and neuron location**
Huang et al.	To examine the role of miR-133b in Mauthner-cell regeneration in zebrafish	• Overexpression of miR-133b inhibited axon regeneration, whereas down-regulation of miR-133b, promoted axon outgrowth • miR-133b regulates axon regeneration by directly targeting *tppp3*, a novel regeneration-associated gene which belongs to Tubulin polymerization-promoting protein family • miR-133b overexpression attenuated mitochondrial motility in M-cells *in vivo*, correlating with enhanced axon regenerative properties	Wild-type, Zebrafish, Hindbrain
Ballout et al.	• To determine the extent to which post-traumatic inflammation following cortical lesion could influence the survival of grafted neurons, • To understand the development of their projections to target brain regions whilst understanding how transplanted cells can modulate host inflammation	• Embryonic motor cortical tissue grafted 1 week after adult motor cortex lesion resulted in an increasing numbers of astrocytes, microglia, oligodendrocytes and hematopoietic cells, compared to implanted grafts at the time of lesion • One week after cortical lesion resulted in more recruitment and activation of inflammatory brain resident mediators and peripheral infiltrating cells compared to day 0 • Graft implantation one week after cortical lesion resulted in (i) increased recruitment of A2 astrocytes in the host transplant and adjacent cortex, (ii) increased oligodendrocytes only within the transplant, and (iii) decreased M1 microglia only within the transplant	Wild-type Mouse, Motor cortex
Chen et al.	• To understand the behavior of Schwann cells migrating into a nerve gap following a transection injury • To reveal their interactions with regenerating axons within the nerve bridge	• After peripheral transection, axonal outgrowth first begins from the proximal stump, followed by Schwann cells migration from proximal and distal nerve stumps • Schwann cells overtake the axonal outgrowth, forming Schwann cell cords within the nerve bridge and most regenerating axons attach to the migrating Schwann cells and follow their trajectory across the nerve gap • Schwann cells play a crucial role in controlling the directionality and speed of axon regeneration across the nerve gap	Wild-type Mouse, Peripheral Nerve
Zeng et al.	Uncover the effects of SIRT1 on IL-33/ST2 signaling and initiation of the inflammatory cascade by TNF-α and IL-1β modulation	• After spared nerve injury, IL-33 and its receptor ST2 were upregulated in sensory neurons in dorsal root ganglia • Intrathecal injections of IL-33 or ST2 antibodies alleviated mechanical allodynia whilst downregulating the expression of TNF-α and IL-1β induced by injury • Reductions of SIRT1 activates IL-33/ST2 signaling and subsequently triggers the TNF-α and IL1β inflammatory cascades that contribute to the mechanical allodynia induced by spared nerve injury	Wild-type Rats, Dorsal Root Ganglia Sensory Neurons
Jara et al.	Determine the mechanisms that contribute to upper motor neuron vulnerability in hSOD1^*G*93*A*^ mice	• Pre-symptomatic hSOD1^*G*93*A*^ mice display altered inhibitory, but not excitatory, circuitry specific to the L2/3 pyramidal neurons in the motor cortex • Exon microarray analysis provides some molecular evidence of altered inhibitory transmission in hSOD1^*G*93*A*^ upper motor neurons • GABA and potassium receptor subunits are differentially expressed in diseased corticospinal motor neurons in hSOD1^*G*93*A*^ mice	hSOD1^*G*93*A*^ Mouse, Motor cortex

The five reviews discuss signaling cascades involving motor neurons, sensory neurons, spinal motor circuits, and electrical stimulation relevant for ALS, SMA, SCI, and regeneration (Table 2). Sobrido-Cameán and Barreiro-Iglesias consider apoptotic signaling cascades after SCI. Shorrock et al. discuss sensory-motor molecular mechanisms in SMA. Eisdorfer et al. contemplate how epidural electrical stimulation can enhance motility after SCI. Alvarez et al. examine the influence of spinal cord circuitry in regeneration of peripheral nerves. Falgairolle and O'Donovan deliberate on the influence of motor circuits on motor neuron vulnerability in ALS and SMA.

**Table 2:** Highlights from the mini- and full- reviews published within this Research Topic.

**Paper**	**Highlights**	**Paper type**	**Disease/Dysfunction model**
Sobrido-Cameán and Barreiro-Iglesias	• Reviews the literature on caspase-8 mediated cell death after spinal cord injury in a variety of animal models • Discusses caspase-8 activated signaling pathways following spinal cord injury • Proposes novel areas to advance the knowledge on the role of caspase-8 and Fas in cell death after spinal cord injury	Mini-Review	Spinal Cord Injury (SCI)
Shorrock et al.	• Highlights that defects in sensory components of the sensory-motor system contribute to motor neuron dysfunction early in SMA • Emphasizes that cell types other than motor neurons play an important role in SMA pathogenesis • Reiterates that therapeutic interventions must rescue the wide array of defects that are observed in SMA	Mini-Review	Spinal Muscular Atrophy (SMA)
Eisdorfer et al.	• Describes the utility of epidural electrical stimulation (EES) in enhancing motility in SCI patients • Identifies several sensorimotor plasticity mechanisms that are considered to be evoked by EES through the activation of peripheral afferents • Evaluates emerging genetic modification tools that modulate afferent fibers to uncovering molecular and circuit mechanisms of EES-induced recovery from SCI	Review	Spinal Cord Injury (SCI)
Alvarez et al.	• Provides a comprehensive conceptual framework to understand how different types of nerve injuries that result in motor neuron axotomy induce distinct regenerative programs that drastically differ in motoneuron preservation, and speed and efficiency of regeneration • Proposes that synaptic plasticity of axotomized motor neurons should be divided into two distinct processes: (1) a reversible, rapid, cell-autonomous, microglia-independent shedding of synapses; and (2) a slower, microglial dependent mechanism that permanently alters spinal cord circuitry • Considers the significance of differential removal of excitatory and inhibitory synapses on synaptic plasticity of axotomized motor neurons	Review	Peripheral Nerve Injury
Falgairolle and O'Donovan	• Discusses abnormalities in the spinal cord circuitry in both ALS and SMA • Describes the sensitivity of the different motor neuron subtypes in both motor neuron diseases • Deliberates on if the selective vulnerability or resistance of different motor neuron types in ALS/SMA can be attributed to their intraspinal connectivity	Review	Amyotrophic lateral sclerosis (ALS) and spinal muscular atrophy (SMA)

Overall, this *Research Topic* describes anatomical, electrophysiological, cellular, and molecular interactions between neural networks and how advancing technologies enable clearer characterizations of dysfunctional neural circuitry.

## Author Contributions

All authors listed have made a substantial, direct and intellectual contribution to the work, and approved it for publication.

## Conflict of Interest

The authors declare that the research was conducted in the absence of any commercial or financial relationships that could be construed as a potential conflict of interest.
